# Transcriptomics in Toxicogenomics, Part III: Data Modelling for Risk Assessment

**DOI:** 10.3390/nano10040708

**Published:** 2020-04-08

**Authors:** Angela Serra, Michele Fratello, Luca Cattelani, Irene Liampa, Georgia Melagraki, Pekka Kohonen, Penny Nymark, Antonio Federico, Pia Anneli Sofia Kinaret, Karolina Jagiello, My Kieu Ha, Jang-Sik Choi, Natasha Sanabria, Mary Gulumian, Tomasz Puzyn, Tae-Hyun Yoon, Haralambos Sarimveis, Roland Grafström, Antreas Afantitis, Dario Greco

**Affiliations:** 1 Faculty of Medicine and Health Technology, Tampere University, FI-33014 Tampere, Finland; angela.serra@tuni.fi (A.S.); michele.fratello@tuni.fi (M.F.); luca.cattelani@tuni.fi (L.C.); antonio.federico@tuni.fi (A.F.); pia.kinaret@helsinki.fi (P.A.S.K.); 2 BioMediTech Institute, Tampere University, FI-33014 Tampere, Finland; 3 School of Chemical Engineering, National Technical University of Athens, 157 80 Athens, Greece; irini.liampa@gmail.com (I.L.); hsarimv@central.ntua.gr (H.S.); 4 Nanoinformatics Department, NovaMechanics Ltd., Nicosia 1065, Cyprus; melagraki@novamechanics.com (G.M.); afantitis@novamechanics.com (A.A.); 5 Institute of Environmental Medicine, Karolinska Institutet, 171 77 Stockholm, Sweden; pkpekka@gmail.com (P.K.); penny.nymark@ki.se (P.N.); grafstromrc@gmail.com (R.G.); 6 Division of Toxicology, Misvik Biology, 20520 Turku, Finland; 7 Institute of Biotechnology, University of Helsinki, 00014 Helsinki, Finland; 8 QSAR Lab Ltd., Aleja Grunwaldzka 190/102, 80-266 Gdansk, Poland; k.jagiello@qsarlab.com (K.J.); t.puzyn@qsarlab.com (T.P.); 9 University of Gdansk, Faculty of Chemistry, Wita Stwosza 63, 80-308 Gdansk, Poland; 10 Center for Next Generation Cytometry, Hanyang University, Seoul 04763, Korea; hakieumy12@gmail.com (M.K.H.); jksakdma0529@gmail.com (J.-S.C.); taeyoon@hanyang.ac.kr (T.-H.Y.); 11 Department of Chemistry, College of Natural Sciences, Hanyang University, Seoul 04763, Korea; 12 Institute of Next Generation Material Design, Hanyang University, Seoul 04763, Korea; 13 National Institute for Occupational Health, Johannesburg 30333, South Africa; natashaS@nioh.ac.za (N.S.); maryG@nioh.ac.za (M.G.); 14 Haematology and Molecular Medicine Department, School of Pathology, University of the Witwatersrand, Johannesburg 2050, South Africa

**Keywords:** toxicogenomics, transcriptomics, data modelling, benchmark dose analysis, network analysis, read-across, QSAR, machine learning, deep learning, data integration

## Abstract

Transcriptomics data are relevant to address a number of challenges in Toxicogenomics (TGx). After careful planning of exposure conditions and data preprocessing, the TGx data can be used in predictive toxicology, where more advanced modelling techniques are applied. The large volume of molecular profiles produced by omics-based technologies allows the development and application of artificial intelligence (AI) methods in TGx. Indeed, the publicly available omics datasets are constantly increasing together with a plethora of different methods that are made available to facilitate their analysis, interpretation and the generation of accurate and stable predictive models. In this review, we present the state-of-the-art of data modelling applied to transcriptomics data in TGx. We show how the benchmark dose (BMD) analysis can be applied to TGx data. We review read across and adverse outcome pathways (AOP) modelling methodologies. We discuss how network-based approaches can be successfully employed to clarify the mechanism of action (MOA) or specific biomarkers of exposure. We also describe the main AI methodologies applied to TGx data to create predictive classification and regression models and we address current challenges. Finally, we present a short description of deep learning (DL) and data integration methodologies applied in these contexts. Modelling of TGx data represents a valuable tool for more accurate chemical safety assessment. This review is the third part of a three-article series on Transcriptomics in Toxicogenomics.

## 1. Introduction

Clarifying the toxic potential of diverse substances is an important challenge faced by scientists and regulatory authorities alike [1]. The rapid generation of genomic-scale data has led to the development of TGx, which combines classical toxicology approaches with high throughput/high content molecular profiling technologies in order to identify deregulated molecular mechanisms upon exposures as well as candidate biomarkers for toxicity prediction [2,3,4,5,6,7].

In the last years, many transcriptomics datasets have been generated to characterize the molecular MOA of chemicals, small molecules and nanomaterials exposure by transcriptomics profiling of the exposed biological systems [5]. At the same time, new ML algorithms have been proposed in order to better understand and eventually predict the genomic behaviour underlying the exposure. Indeed, TGx datasets have been exploited in the context of drug repositioning [8,9], toxicity prediction [10,11,12,13], definition of adverse outcome pathways (AOP), and as a valuable source to develop new approach methodologies [14,15].

Notably, TGx approaches have been used to analyze quantitative transcriptomic data, to determine the BMD and estimate the critical point of departure for human health risk assessment [16,17,18,19]. These approaches are applied in the framework of read-across analysis, with the aim of predicting the behaviour of uncharacterized compounds by comparing them to other substances whose molecular effects are known [20,21].

TGx shifts the focus from traditional end-point-driven analysis to a systems biology approach, allowing to better understand and predict the alterations in the molecular mechanisms leading to toxicity. In this context, different methods for the study of gene co-expression networks is of great interest to identify common patterns of expression among relevant genes [2,3,4,5,6,22].

Furthermore, different ML methodologies have been developed and applied to the analysis of TGx datasets for the purpose of identifying toxicogenomic predictors. ML includes both unsupervised and supervised methods. The unsupervised methods, such as clustering, do not require any prior classification of the samples, grouping them based on similarities of selected features. On the other hand, supervised methods require discrete or continuous endpoints. They are often combined with strategies to identify an optimal subset of features that can discriminate the endpoint values. This subset of features can then be used for the prediction of the class or the effect of a new sample. A wide range of algorithms has been proposed to build robust and accurate predictive models, including linear and logistic models, support vector machines (SVM), random forests (RF), classification and regression trees (CART), partial least squares discriminant analysis (PLSDA), linear discriminant analysis (LDA), artificial neural networks (ANNs), matrix factorization (MF) and k-nearest neighbours (K-NN) [23,24,25,26]. Classic techniques such as linear and logistic models have been the first to be applied in such modelling tasks and can still be considered the methods of choice, especially when analyzing small datasets. More recently, novel methodologies based on artificial intelligence (AI) and deep learning (DL) have been used with great success in a wide range of applications, including image analysis, and also for the development of TGx-based predictive models [27]. These new approaches are envisaged to produce more accurate predictions and open new horizons to the identification of biomarkers with discrimination performance and predictive ability [5,28]. One of the biggest challenges faced in TGx is the limited amount of samples in the available data, especially in specific fields such as nanotoxicology. ML techniques can be very useful to overcome small sample sizes in TGx studies by combining several gene profiles from multiple related biological systems or by applying transfer learning techniques [27].

In this review, we present the state-of-the-art methodologies developed to interpret, analyze and model already preprocessed omics data. These include BMD analysis, gene co-expression network and ML methods for predictive modelling. We discuss the main ML methodologies, highlight scenarios where each methodology is most suited, the pros and cons of the different approaches, and which are the best validation strategies. We also provide a brief overview of data integration methodologies for multi-omics data analyses.

## 2. Benchmark Dose Modelling

One of the main goals of toxicity assessment is the study of exposure–response relationships that describe the strength of the response of an organism as a function of exposure to a stimulus, such as chemical exposure, after a certain time. These relationships can be described as dose-response curves where the doses are represented on the x-axis and the response is represented on the y-axis. From these curves, a BMD value is calculated as the dose (or concentration) that produces a given amount of change in the response rate (called BMR) of an adverse effect. Normally, the BMR value is 5% or 10% change in the response rate of an adverse effect relative to the response of the control group. Furthermore, estimations of the lower and upper confidence interval for the BMD value are also computed and are called BMDL and BMDU, respectively [29,30,31].

In the last years, dose-response studies have been integrated with microarray technologies, thus introducing gene expression as an additional important outcome related to the dose. Indeed, the genes whose expression changes over the dose are of particular interest, since they provide insights into efficacy, toxicity and many other phenotypes. A specific challenge is to identify genes with expression level changing according to dose level in a non-random manner, identifiable as potential biomarkers [32].

The combination of microarray technology with BMD methods results in a bioinformatic tool that provides a comprehensive survey of transcriptional changes together with dose estimates at which different cellular processes are altered, based on a defined increase in response [33]. A classic BMD modelling pipeline involves fitting the experimental data to a selection of mathematical models, such as linear, second- or third-degree polynomial, exponential, hill, asymptotic regression, Michaelis–Menten models etc. Among all, the best model is selected by using a goodness of fit criteria, such as the Akaike information (AIC) or the goodness-of-fit *p*-value.

A predefined response level of interest, called BMR, is identified and the optimal model is used to predict the corresponding dose (BMD) [34]. Moreover, the European Food Safety Authority (EFSA) suggests reporting both the lower and upper 95% confidence limit on the BMD [35]. The most popular tool to perform BMD analysis is BMDS (Table 1), which is developed by a U.S. Environmental Protection Agency’s publication [29]. It implements the following pipeline: first, the BMR value is selected. A set of appropriate models and their parameters for which the model fit are assessed. Then, the BMDs and BMDLs values for the adequate models are estimated. The optimal values coming from the model with the lowest AIC are selected.

BMD approach is also implemented by the PROAST software (https://www.rivm.nl/en/proast) (Table 1), developed by the Rijksinstituut voor Volksgezondheid en Milieu institute (RIVM). Although RIVM and EPA aim to achieve consistency between these tools, there are still differences in some of their default settings and functionalities [36,37]. For example, PROAST allows for the statistical comparison of dose-responses between subgroups (covariate analysis) and offers larger flexibility in plotting [37,38]. PROAST can be run as a library in R, but also using two web applications that offer only basic functionalities for quick access (https://efsa.openanalytics.eu/, https://proastweb.rivm.nl/).

A similar pipeline for toxicogenomics applications is implemented in the java-based US National Toxicology Program’s BMDExpress 2 tool, where a dose-response model is fitted for every gene, whose expression value is the response variable for the different doses [39,40,41] (Table 1). Furthermore, an R package for the dose-response analysis of gene expression data, called ISOgene has been proposed [42,43]. It implements the testing procedure proposed in, in order to identify a subset of genes showing a monotone relationship between the expression values and the doses [44]. This tool does not compute any BMD or BMDL values but returns a set of genes with a statistically significant monotone relationship (Table 1).

More recently, a novel user-friendly software based on R/Shiny, BMDx, has been introduced [31]. In addition to the evaluation of dose-response of each gene expression pattern, BMDx also provides ways to compare multiple exposures or multiple time points along with suggesting functional characterization of the identified dose-response genes (Table 1).

## 3. Gene Co-Expression Network Analysis

Gene co-expression network analysis is a systems biology method used to describe the correlation patterns among genes across different experimental samples. It allows representing, investigating and understanding the complex molecular interactions within the exposed system [22,45]. The genes and their interactions are represented as a network (or graph) where the genes are the nodes of the network and their strength of similarity is represented as weighted edges between the nodes [46].

To understand the nature of cellular processes, it is necessary to study the behaviour of genes by means of a holistic assessment [47]. Thus, the inference of gene co-expression networks is a powerful tool for better understanding gene functions, biological processes, and complex disease mechanisms. Indeed, co-expression network analysis has been widely used to understand which genes are highly co-expressed within certain biological processes or differentially expressed in various conditions. They are also used for candidate disease-related gene prioritization [48], for functional gene annotation and the identification of regulatory genes [49]. For example, Kinaret et al. systematically investigated the transcriptomic response of the THP-1 macrophage cell line and lung tissue of mice after exposure to several nanomaterials by using a robust gene co-expression network inference method [2,50]. Subsequently, they ranked the genes in the network by computing different topological measures, identified and functionally characterized a set of genes that play a key role in the adaptation to exposure. Other approaches focus on identifying gene network modules associated with specific patterns of drug toxicity [45].

Studying the topology of the gene co-expression network allows identifying communities of genes that show similar behaviour. Moreover, the use of centrality measures facilitates the identification of genes that are hubs in the network [49]. A classical analysis performed on inferred gene co-expression networks is the identification of functional modules, such as groups of co-expressed genes. This is usually carried out by means of standard clustering algorithms, such as k-means, hierarchical clustering, spectral clustering, or by means of community detection algorithms [51]. The clustering method needs to be chosen with consideration because it can greatly influence the outcome and meaning of the analysis [49]. Modules can subsequently be interpreted by functional enrichment analysis, a method to identify and rank overrepresented functional categories in a list of genes.

### Algorithms to Infer Gene Co-Expression Networks

The first challenge in this type of analysis is the identification of the best algorithm used to infer the gene co-expression network. Indeed, starting from the preprocessed TGx data, different methods to construct the gene co-expression network can be applied [52,53,54]. These methods differ on how they calculate the similarities between the expression profiles and how they remove the non-relevant connections. The dependence between the expression profiles is usually computed by means of information-theoretic methods such as the pairwise correlation coefficient and mutual information (MI) [45,55]. The main difference between the two methods is that the first one is able to identify only linear dependence between the profiles, while the second is also able to identify non-linear dependencies.

Various algorithms have been proposed based on information theory. Some of the most important ones are RelNet [56], ARACNE [57], CLR [58], PANDA [59] and WGCNA [60]. RelNet works on two steps: it first creates a completely connected gene co-expression matrix where the mutual information between all genes is computed [56]. Subsequently, a threshold is defined, called TMI, that identifies which are the associations to be considered as significant. ARACNE computes the mutual information for all gene pairs of a gene expression dataset and excludes all the mutually independent gene pairs. Consequently, the ARACNE algorithm reduces the number of false positives connections, by cutting the less strong association between every triplet of genes in the network [57].

The CLR algorithm is an extension of the relevance network, but there is a correction step to eliminate false correlation and indirect effects [58]. Similarly to RelNet and ARACNE, this algorithm uses the matrix of MI values between all regulators and their potential target genes. In the next step, the CLR calculates the statistical likelihood of each MI value within its network context. This algorithm compares MI values of gene pairs with the background distribution of MI values. The interactions whose MI scores stand significantly above background distribution of MI scores are considered as the most probable interactions. This step eliminates many of the false correlations in the network (e.g., when transcription factors co-vary weakly with a lot of genes or a gene co-varies weakly by transcription factors of different factories).

Unfortunately, applying different methods to the same omic dataset may not always result in consistent co-expression networks. For this reason, Marwah and collaborators recently proposed a tool, called INfORM (Inference of NetwOrk Response Module), able to infer a more stable and robust network by applying an ensemble strategy [50]. INfORM derives gene networks by employing a two-level ensemble strategy that combines models proposed by multiple network inference algorithms (ARACNE [57], CLR [58], MRNET, MRNETb [61]), to ensure the robustness of gene–gene associations. Network topology information and user-provided biological measures of significance (e.g., differential expression scores) are used together to obtain a robust rank of genes in the network, by means of the Borda method. Community detection methods are used to identify modules of closely correlated genes. Such modules are characterized by the importance of member genes in the network and GO enrichment is performed for functional characterization. Finally, the user can assess the characteristics of the modules and the functional similarity between modules to define the response module that represents the best network properties. The biological significance of this response module can be inferred from the summarized representation of enriched GO annotations clustered by their semantic similarity.

A different approach is to infer directed graph networks, which allow not only to reveal the systematic coordinated behaviour of sets of genes but also the identification of causal and regulatory relationships between them [61]. These models can overcome the pitfalls of correlation networks, which are sensitive to technical and biological noise and may produce artefacts due to the loss of the direction of the correlation between each pair of genes during the network construction [62,63,64,65]. There are a number of methods for learning directed acyclic graphs (DAGs) available [66], but significant effort is being made to modify them in order to produce meaningful results by analyzing high-dimensional omics data [62,63,64,65,66,67].

## 4. Read-Across

The main assumption of read-across studies is that structurally similar compounds are likely to share a similar toxicological profile. These approaches are used to fill toxicological data gaps by relating to similar chemicals for which test data are available [68]. Traditional read-across studies rely only on the similarities between the chemical structure of the compounds. Different measures have been proposed to compute the chemical structure similarity and also multiple tools for read-across, mainly based on the nearest neighbour algorithm, have been developed [69,70]. However, these approaches are limited to the fact that the chemistry cannot explain the complex biological processes that are activated by substance exposure [71].

TGx datasets, such as DrugMatrix [72], Connectivity Map (CMAP) [73] and LINCS 1000 [74], can be used to profile the biological fingerprint of multiple chemicals and allow to compare the measured compound with a huge number of tested chemicals at the transcriptomic levels. Thus, the assumption underlying related read-across studies could be that if two chemicals have similar biological profiles they have a similar adverse outcome. Biological-based read-across could be complemented to the structure-based read-across.

For example, Zhu et al. developed a read-across method based on a consensus similarity approach starting from different biological data, to assess acute toxicity in the form of estrogenic endocrine disruption [68]. Moreover, Serra et al. proposed a network-based integrative methodology to perform read-across of nanomaterials exposure with respect to other phenotypic entities such as human diseases, drug treatments and chemical exposures [20]. In particular, they integrated gene expression data from microarray experiments for 29 nanomaterials, with other gene expression data for drug treatments and data available from the literature that relates differentially expressed genes to chemical exposures and human diseases. They created an interaction network that was used to contextualize the effect of the nanomaterials exposure on the genes by comparing their effects with those of chemicals and drugs with respect to particular diseases. With this approach, the authors identified potential connections between metal-based nanoparticles and neurodegenerative disorders.

Furthermore, the toxFlow web-based application is available to perform read-across and toxicity prediction by integrating omics and physicochemical data [21]. The implemented workflow allows to filter omics data with enrichment scores and then merge them together with the physicochemical data into a similarity-based-read across method to predict the toxicity level of a substance by inferring information from its most similar ones. However, the user still needs to define an initial grouping/read-across hypothesis regarding the variables that will be considered important and the threshold values, that set the boundary to the neighborhoods of similar ENMs. Apellis web application updates the toxFlow methodology by automating the process of searching over the solution space in order to find the read-across hypothesis that produces the best possible results in terms of prediction accuracy and number of ENMs for which predictions are obtained [75]. To do so, a stochastic genetic algorithm that serves the selection of both the appropriate variables and the threshold values simultaneously was developed and is trained during the first step of the procedure, while the predicted toxicity endpoint is retrieved during the second part of it [75].

## 5. Adverse Outcome Pathways

Adverse outcome pathway (AOP) is a conceptual framework that couples existing knowledge on the links between a molecular initiating event (MIE), such as contact of nanomaterial with Toll-like receptors on the cell surface, with the activation of a chain of causally relevant biological processes or key events (KE), e.g., the production of inflammatory cytokines, with the resulting adverse outcomes (AO) at the level of the organ or the organism (e.g., lung fibrosis) [76,77]. Coupling of gene expression profiling with bioinformatics-driven placement of the results into AOP descriptions has the potential for quantitative analysis of adverse effects that combines in vitro-derived mechanistic analyses with causally relevant modes-of-action and related key events [77,78]. As AOPs can span different cell types, numerous in vitro assays may need to be associated with a single one [14,76]. The details of the coupling are still being worked out by the community but mapping the results of pathway analyses to KEs is a simple alternative. For example, if the bioinformatics results cover all of the KEs in the chain leading up to an AO, then the AOP could be considered as active. The point-of-departure concentration might be defined as the lowest concentration where all of the KEs are activated. Naturally, this depends on the type of model systems used and its limitations (see Part I of this article series). However, early activation of molecular mechanisms in vitro have been shown to be predictive of phenotypic effects taking place later or in other systems [12], providing a basis for use of TGx data to cover largely all information blocks in an AOP [78]. Pathway annotations that can be considered include the WikiPathways, Molecular Signatures Database, KEGG, Reactome, Gene Ontologies Biological Process but also the Predictive Toxicogenomics Space (PTGS) components and other dedicated descriptions of toxicity pathways [12,79]. A more refined POD concentration could be provided by coupling the aforementioned AOP-based transcriptomics data analysis with BMD modelling to evaluate the dose-response nature of the exposure to ENMs at gene or pathway level. Subsequently, these transcriptional BMD values may be used to rank the potency of the nanomaterials to induce changes related to specific adverse outcomes of interest at the lower levels of biological organization, and group them according to the severity of the biological effects they cause [18].

## 6. Machine Learning in Toxicogenomics

ML methods have significantly advanced in recent years and are proven to be important alternatives to experimental testing for chemicals and nanomaterials [80,81,82]. The value of TGx-derived biomarkers of toxicity lays in the fact that they can be detected earlier than histopathological or clinical phenotypes [83]. The development of ML methods and tools for omics data analysis has also been proposed and several algorithms have been successfully applied to the analysis of omics data also in the TGx field [84]. For example, Rueda-Zarate, Hector A., et al. proposed a strategy that combines human in vitro and rat in vivo and in vitro transcriptomic data at different dose levels to classify the compound toxicity levels [85]. They combined machine learning algorithms, with time series analysis by taking into account the genes correlation structure across the time. Furthermore, Su et al. developed a drug-induced hepatotoxicity prediction model based on biological feature maps and multiple classification strategies [86]. They use a biology-context based gene selection to identify the most discriminative genes and showcased their methodology on the Open TG-GATEs and DrugMatrix datasets.

ML algorithms use data-driven approaches to develop predictive models. Data derived from empirical experiments is first analysed to assess its quality, and, if necessary, it is preprocessed to improve the stability of the ML models. Common preprocessing techniques include filtering out features that are not informative, removing anomalous observations (outliers, noisy data) or filling data gaps. After this step, the dimensionality of the training data can still be excessive, so the data can be further preprocessed using dimensionality reduction techniques.

**Figure 1:**
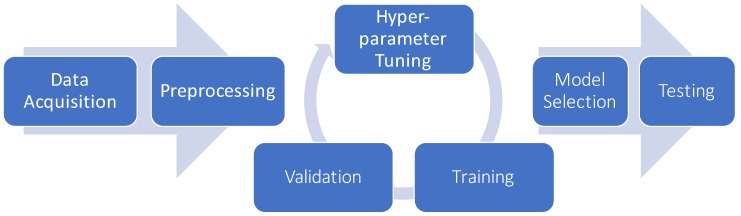
Example of ML pipeline for TGx data. Data Acquisition and Preprocessing: Data is collected and analyzed to ensure the quality of the dataset. During the preprocessing, feature selection and/or feature transformations may be applied to improve stability. Training-Hyperparameter tuning-Validation loop: candidate models are fit to the data. This is embedded in an iterative process where for each candidate model the best hyperparameters are optimized through the validation step. Model Selection and Testing: Optimized candidate models are identified and the best ones are tested on a final hold-out dataset to evaluate generalization capabilities.

The preprocessed data is used to train ML models that can predict a variable of interest (supervised learning) or detect patterns in the dataset (unsupervised learning). In addition to estimating parameters fitted to the data, most models also provide a set of hyper-parameters that must be optimized to achieve best performances, like the number of clusters in k-means, or the number of trees in a random forest. After training, the capability of the model to generalize beyond the training data is evaluated on an independent and identically distributed test set. In the case of multiple competing models, the best model of each family of predictors is evaluated and the optimal model is deployed in the real-world environment. A graphical representation of the process is shown in Figure 1.

### 6.1. Dimensionality Reduction and Feature Selection

Since TGx data usually present a large number of measured molecules compared to the number of samples, they can suffer from the curse of dimensionality. Thus, the model overfitting, the spurious correlations and a trade-off between accuracy and computational complexity have to be taken into account when modelling these data [87].

Dimensionality reduction techniques and feature selection methods can mitigate these issues and can be used in combination with ML approaches to build predictive modeling [24]. Some examples of dimensionality reduction techniques are principal component analysis (PCA) [88], multidimensional scaling (MDS) [89], t-distributed stochastic neighbour embedding (t-SNE) [90] and Uniform Manifold Approximation and Projection (UMAP) [91].

Probabilistic component modelling can be a powerful technique as it combines dimensionality reduction with highly interpretable ML models [10,92]. It can be used in unsupervised mode to identify response modules that describe aspects of cellular response to chemicals or in a supervised way to predict responses based on omics input data. The Predictive Toxicogenomics Space (PTGS) scoring concept tool is based on modules derived from Latent Dirichlet Allocation (LDA) probabilistic component model analyses of the entire CMAP dataset [73] of over 1300 chemicals and drugs. To begin, 100 response components were derived. Expression data was then integrated with the NCI-60 DTP cellular screening database (222 chemicals) to identify 14 components that corresponded with cytotoxicity at 50% growth-inhibitory level or above. Further ML assessment selected 4 of these 14 components that were able to predict liver toxicity. The multi-view Group Factor Analysis (GFA) and Bayesian Multi-tensor Factorization (MTF) are in some ways more advanced versions of probabilistic component modelling but also have their own limitations, so decisions on their use have to be made individually.

If the goal of the analysis is to reduce the dimensionality by preserving the original features, feature selection approaches can be a better alternative. Indeed, it allows to reveal significant underlying information and to identify a set of biomarkers for a particular phenotype [24]. Examples of these are filter approaches such as information gain, correlation feature selection (CFS) [93], Borda [94], random forests [95,96], FPRF [26], and Varsel [97]. More advanced modelling based on genetic algorithms, such as GALGO [98] and DIABLO [99], GARBO [100], allows taking into account the non-linear correlations between candidate biomarkers.

Feature selection methods for the identification of biomarkers of toxicity can be used in combination with ML approaches to predict the toxicity of different drugs and chemicals [12,101,102]. For example, Eichner et al. [103] applied an ensemble feature selection method in conjunction with bootstrapping technique to derive reproducible gene-signature from microarray data for the carcinogenicity of drugs. The application of stable feature selection methods is particularly important since it may accelerate the screening for promising candidates and hence have more efficient and less costly processes for drug development.

Su et al. proposed a multi-dose computational model to predict drug-induced hepatotoxicity based on gene expression for toxicogenomics data [104]. Their methodology is based on a hybrid feature selection method, called MEMO, which uses the dose information after drug treatment based on a dose response curve to deal with the high-dimensional toxicogenomics data after. They validated their model using the Open TG-GATEs database and they show that the drug-induces hepatotoxicity can be predicted with high accuracy and efficiency.

#### Stability and Applicability Domain

TGx data are the result of an experimental measure that is prone to both technical and biological noise, due to the complexity of the exposed system. Thus, stability and reproducibility play a key role in the analysis [105]. For example, multivariate methods can identify different subsets of candidate biomarkers with equal or similar accuracy, even if the feature selection algorithms are used on the same data [26,106,107]. Other challenges that should be taken into account when creating models from TGx data involve the applicability domain (AD) of the predictors, the number of predictors in the models.

According to the OECD principle of validation [108], one of the essential steps in model implementation is the definition of the AD. Indeed, predictions extrapolated outside of the model’s AD may be less accurate [109]. Even though different methods have been proposed to compute and evaluate the model AD, there is still a lack of a uniform definition. One of the most common methods to compute AD is based on the leverage methods such as the Williams plot [110]. This method can be used to compute AD for linear regression models and is also useful to identify outliers in the data. Other methods can be the standardization approach [111] and the euclidean and city block distance methods [112]. The AD of non-linear models can be computed with kernel-based estimators or k-nearest neighbours method [112]. The AD for classification models can be computed by using the PCA-based and range-based methods [113].

Recently, a new methodology for feature selection from complex data, called MaNGA has been proposed [114]. MaNGA uses a multi-objective optimization strategy to identify the minimum set of predictive features with the widest AD, better predictivity capability and high stability. Even though the MaNGA strategy has been implemented for the development of robust and well validated predicting QSAR models, it could be easily applied to identify biomarkers of toxicity from TGx data.

### 6.2. Clustering

Clustering is an unsupervised learning exploratory technique, that allows identifying structure in the data without prior knowledge on their distribution. The main idea is to classify the objects based on a similarity measure, where similar objects are assigned to the same class [51,115,116]. Transcriptomic data are characterized by a huge number of features (genes), thus the first step in gaining some understanding of microarray data is to organize them in a meaningful way. Cluster analysis has been used as an extremely helpful method to analyze and visualize this data. The main objective of performing cluster analysis with transcriptomic data is to group together genes that share the same pattern of expression but differ from the genes in other clusters. The main assumption is that the genes in the same cluster may be involved in similar or related biological functions [117,118,119].

Different clustering algorithms are available and some well known algorithms are listed in Supplementary Table S1. Some entries (e.g., biclustering) refer to families of algorithms stemming from a common structure. Different clustering algorithms can produce different results starting from the same input data, and even a single algorithm may produce two different results in two different runs if featured with a random component. Different metrics, such as the Davies-Bouldin index, the Dunn index, and the silhouette index [120], can be used to internally validate the clustering results based solely on the data on which clustering was made. Other metrics, such as the Rand index, the Jaccard index, the Normalized Mutual Information, and the F-measure [121], can be used to compare different clustering results. In order to improve the stability of an algorithm with a random component, consensus clustering can be used to aggregate the results from a number of runs. It can also be used in case an ensemble approach is desired [122].

Furthermore, due to the intrinsic complexity of the omics technologies and processes involved, experiments can also produce low quality data. As in other domains, the presence of outliers may strongly influence the results. Various methods exist to detect outliers and to handle them. When an outlier is detected it may simply be removed, but also other approaches are possible like substituting it with an object more similar to the others, or assigning a reduced weight [123,124].

Applications of clustering in TGx include evidencing groups of samples/experimental conditions by similarity in gene expression or, analogously, classes of genes by similarity in their expression between samples/experiments. If the focus is on the responses to different stimuli, fold change similarities between pairs of readings occurred before and after the stimuli may be used as a similarity measure. Clustering may be applied to data before training a predictive model in order to tune the stratification of the samples or to train different models on different subpopulations [125]. McNicholas and Murphy [126] applied k-means, PAM, hierarchical, and mixture models to map the correlation between gene expression levels in data collected from two different studies about leukaemia and colon cancer. The clustering algorithms were compared using the Rand index, resulting in a better performance of the mixture models. Gao et al. [122] applied consensus clustering to transcriptomics time series data from *E. coli* subjected to toxicants at various dosages. Self-organizing map, a kind of ANN producing dimensionality reduction to a discrete space, was used as the underlying algorithm for the consensus clustering. The resulting clusters were mostly consistent with prior toxicological knowledge. Nystrom-Persson et al. [117] applied hierarchical clustering (Ward’s method with Pearson distance) to the toxicogenomics database Open TG-GATEs to study the hepatotoxicity of pirinixic acid. Hasan et al. [127] applied a number of hierarchical clustering configurations to the Japanese Toxicogenomics Project dataset to detect toxic DDs and their associated biomarker genes. They concluded that Ward’s method with similarities computed with Minkowski distances produced the better results.

### 6.3. Classification

In supervised learning, classification is the task of predicting the class to which a sample belongs, given the class of previously seen samples. To build such a predictive system, it is necessary to provide as training samples to a learning algorithm both the TGx features and the toxicity label. In classification tasks related to TGx data, it is common to assign to the positive class the “interesting” effects, which, for example, may correspond to events of toxicity. Different classification algorithms have been applied to the problem of predicting toxicity in the liver and kidney using TGx features, in vitro endpoints and molecular descriptors [128,129,130].

For example, Minowa et al. [130], proposed a methodology for the prediction of future kidney injury based on gene expression data measured at most 24 h after a single exposition. Specifically, kidney gene expression sampled at different time points (3 h, 6 h, 9 h, 24 h) after administration of a single dose was used to predict proximal tubular injury in rats for up to 28 days of repeated doses administration. The authors trained several linear SVM models on gene expression data at different time points. The best model, according to sensitivity (93%) and selectivity (90%), have been obtained using 19 differentially expressed gene features at 24 h after administration of a single dose. In Low et al. [128], several models have been employed to build rat hepatotoxicity predictors based on QSAR methodologies as well as toxicogenomics features. Gene expression from rat kidney after 24 h from single exposure were used together with molecular descriptors to train different models, namely kNN, SVM, RF and distance weighted discrimination (DWD) in order to predict in vivo hepatotoxicity events. All the models were trained using a five-fold cross validation procedure. Each model has been trained using only molecular descriptors, transcriptomics data and a combination of the two. Even though hybrid QSAR-toxicogenomics models had comparable performances with predictors built with only toxicogenomics features, post-hoc analysis of how these types of features interact can help to identify relevant transcripts and chemical alerts for hepatotoxicity.

When training a model, the whole dataset is split into training data and test data. The training data is used to fit the model, and the test data is used to evaluate the quality of the fitted model against data unseen during training. Once fitted on the train data, the model’s generalization capabilities are estimated by multiple metrics, such as the accuracy and F1-score that are computed starting from a confusion matrix [131].

The objective is for the model to adapt to the training data enough to be able to generalize to new data samples, but not too much to overfit and being unable to generalize to new samples. Overfitting is particularly worsened in cases where the number of samples is smaller than the number of features so that the joint distribution cannot be properly represented by the data [132]. Many methodologies have been proposed to dampen this effect by prioritizing the most predictive features, e.g., Liu et al. [133], proposed a ranking algorithm for the prioritization of predictive features based on an iterative sampling scheme. At each iteration, a random subset of features, smaller than the number of samples, is chosen, and used to train a classification model. For each feature, the predictive performances are registered and when the ranking stabilises, the algorithm ends. The efficacy of the algorithm was validated on a toxicogenomics database where the top predictive genes resulted functionally related to several phenotypes of liver toxicity.

The more a dataset is unbalanced, the less informative some evaluation metrics become. This is because, during training, every classifier learns with less effort the negative class, at the expense of a rising rate of false negatives. Consider, as an extreme example, a dataset made of 99% negative samples and 1% positive samples. A trivial classifier that always outputs the negative label, would have an accuracy of 99% but is essentially useless.

Different methodologies can be applied to compensate for imbalances in the dataset. During training, the dataset can be resampled, i.e., to reduce the imbalance in the training data, the majority class can be down-sampled (discard a given proportion of the samples), the minority class can be over-sampled, or both. Over-sampling can be as simple as randomly adding duplicate samples, or it can be a generative scheme that creates new synthetic samples combining the actual samples like hybrid methods, such as ROSE [134,135], and SMOTE or its variants [136,137]. Also, it is worth noting that these methods are used only to stabilise model fitting, meaning that resampling schemes are performed only on the training dataset. Since the test dataset is used to evaluate the generalization capabilities of the model simulating new, unseen data, it is important that the test data distribution is not altered by resampling.

Another approach to reduce the effects of imbalance in the dataset is to weight differently classification errors of the classes, e.g., a false negative is ten times worse than a false positive. A trivial weighting scheme is to assign to each class the inverse of the corresponding class proportion as a weight. In conjunction to either approach or a combination of the two, after training, model evaluation should be performed using a metric that takes into account the proportions of each possible outcome such as the Matthews Correlation Coefficient [138,139].

### 6.4. Regression

Regression is a supervised learning methodology that estimates the relationship between the features (a.k.a. independent variables) and a continuous variable referred to as outcome or dependent variable. It is used in TGx to predict important quantities as the level of toxicity of a compound, the half maximal inhibitory concentration (IC50), the survival, or differences between in vitro and in vivo response.

The simplest regression algorithm is the linear regression, where the outcome is predicted by a linear function of the features. Also, nonlinear regression methods are available, where the outcome depends on the features by a more complex function. E.g. Schüttler et al. [140] used the nonlinear Hill equation to describe the time- and concentration-dependent fold change after compound exposure in zebrafish embryo microarray data. Farmahin et al. [16] applied exponential 4, exponential 5, Hill, power, polynomial, and linear models, choosing the best fitting one, to predict the BMD for various types of cancer from microarray gene expression data of rats exposed to different chemicals and doses, measured at 4 time points.

ANNs are well known techniques for nonlinear regression [141]. While sometimes avoided for their black-box nature, they can offer high predictive performances. Deep approaches are viable and have been applied with varying results to predict toxicity. They are promising but not always provide better results than shallow techniques because of the small quantity of data available with respect to other application domains like computer vision or spoken language recognition [142]. For a description of deep learning see Section 6.5.1.

Since using a high number of features produces less compact models and possibly overfitting, a variety of techniques allows to select or reduce the features. Previous external knowledge can be used to select features [143], or they can be algorithmically combined to get more synthetic ones (e.g., with PCA as with a type of unsupervised ANNs called autoencoders [144,145,146]) or part of them can be removed by a preprocessing step (e.g., with a minimum redundancy maximum relevance selection process [147]). Another approach consists in penalizing the weights of the features, possibly down to 0, when they are in fact removed. There are algorithms that apply these penalizations during the fitting of the model. The penalization may be linear in the value of the parameter, as in LASSO models [148], quadratic in the value of the parameter, as in ridge regressions [149], or a combination of the two, as in elastic nets [150].

After a regression model has been trained, it is necessary to internally and externally validate it. Internal validation is performed on the same data that was used in training. It measures how well the model fits the original data, but does not measure overfitting. External validation is performed on a different dataset than the one used for training. Most of the quality measures can be used for each kind of validation just changing the data the model is applied to. Prediction quality measures are extremely variegated, as potentially any similarity measure between the model outputs and the correct outcomes can be used. There are measures of distance between the model output and the correct output, like the root mean square error (RMSE) or the mean absolute error (MSE), measures of goodness of fit, correlation, collinearity, ordering, or other aspects, e.g., the coefficient of determination $R^{2}$ or the $q^{2}$ [151]. The best choice for a quality measure depends on the domain of application of the model, i.e., on how the errors impact the utility for the user.

Regression, as clustering, may be affected by outliers (see Section 6.2).

### 6.5. Model Selection and Hyper-Parameter Optimization

In order to find the best trade-off between model complexity and data availability, it is useful to train more than one model and compare their performances on the test dataset. In addition to model parameters most of the models have another set of hyper-parameters that need to be tuned to achieve optimal performances, like the number of neighbours in k-NN, the number of trees in a random forest or the number of layers, the number of units and the activation functions in a neural network.

These hyper-parameters cannot be inferred directly from data like other training parameters, and need to be estimated by means of an explicit search in some parameter space. Care must be taken when performing hyperparameter optimization since models cannot be evaluated neither on the training set nor on the test set to avoid producing over-optimistic error estimates. The solution is to split the data into three datasets namely, training, validation and test sets [132]. A common rule of thumb is to use 65% of the samples for training, 15% of the samples for validation and 20% of the samples for testing.

When there is not enough data to ensure that model parameter estimates and performance estimates are stable, a simple split of the data is not the most efficient use of data. A more data-efficient approach is k-fold cross-validation, in which the dataset is randomly split into k subsets of approximately the same size, then iteratively, one of the k subsets is used as a validation set and the remaining k-1 subsets as training. The cross-validated estimate is then the average across the k runs, common choices for k are 5 or 10. The limit case where k is equal to the number of samples is called leave-one-out cross-validation.

In case of classification, care must be taken during either type of splitting in ensuring that the class distribution is preserved across the splits. This is particularly important for the case of heavily imbalanced datasets, where some of the splits may completely miss the less represented classes. To overcome this problem, the split may be performed taking into account the class labels and ensuring that each, e.g., the validation set has the same proportion of samples from each class.

Modeling of toxicogenomics data should always take into account the issues. For example, Minowa et al. identified genomic biomarkers for drug-induced renal injury from gene expression data, by applying filter based feature selection and linear classification algorithms. They evaluated their model by using a five-fold cross validation strategy and achieved high sensitivity and selectivity. The genes included in their model were primarily involved in DNA replication, cell cycle control, and oxidative stress and chemical stimuli.

More recently, Furxhi et al. [102], compared the performances of different classifiers to predict nanomaterials in vitro toxicity. They used physicochemical properties and in vitro experimental conditions, from the safe and sustainable nanotechnology (s2NANO) database to predict the toxicity of nanomaterials based on cell viability. Their comparative analysis included eight classifiers of different categories such as rule induction, decision trees, function-based and Bayes classifiers. Furthermore, they used a meta-classifier approach to combine all their results. To train and validate the different models, they split the S2NANO dataset into a training (60%) and validation (40%) set. The training set was heavily unbalanced (with only a few toxic samples), thus they use the SMOTE technique to oversample the minority class. The internal validation was performed by using a 10-fold-cross validation strategy to reduce the randomness of the results, while the validation set was used for external validation. Eventually they used the Copeland index to identify the optimal classifier, that was an ensemble of random-forest, locally weighted learning and k-nearest-neighbour using euclidean distance classifier.

#### 6.5.1. Deep Learning

In the ML field particular attention has been given to DL methodology as a very good alternative for big data analytics with a high rate of success [152]. This rapid advancement has been due to the development of more powerful GPU hardware, automatic differentiation software and the development of new architectures based on the ReLU activation function that reduced the issue of the vanishing gradient [153]. DL methods are composed of multiple processing layers and are able to cope with a high level of abstraction [154]. One of the biggest differences between DL methodologies and classical shallow learning is that DL does not necessarily require a feature extraction step before the learning process [154]. Indeed, DL methods take advantage of their multilayer structure to extract abstract and sophisticated features from the raw data input during the training process.

Algorithms for DL that have been used with success [155] include feedforward neural network (FNN) [156], convolutional neural network (CNN) [157], and graph convolutional network (GCN) [158]. For example, Wang et al. [159] compared the performances of deep neural networks (DNN) with respect to RF and SVM in the prediction of chemically induced liver injuries. They used whole-genome DNA microarray data to predict the presence or absence of three endpoints (biliary hyperplasia, fibrosis, and necrosis) for the drugs in the Open TG-GATEs database and DrugMatrix. The datasets were strongly unbalanced, with lots of negative samples and few positive ones, so they applied the SMOTE algorithm and used multiple metrics, such as F1 and MCC, to evaluate the model performances. Their results show that DNNs have better performance than SVM and RF with a higher generalization capability on the phenotype prediction.

The increasing interest in DL also favoured the creation of a high number of frameworks and platforms for the development of custom applications. Among those, the most used open source frameworks are Tensorflow, PyTorch and MXNet. Apart from frameworks, also platforms for the execution of computational experiments have become common. Many companies nowadays provide the hardware infrastructure required to train DL models as cloud instances in which is possible to develop “notebook” style general applications like Google Colab, Amazon Sagemaker and Azure Notebooks, as well as commercial platforms specialized on the analysis and integration of TGx data such as the Enalos Analytics (http://enalossuite.novamechanics.com) that is used by NanoSolveIT H2020 nanoinformatics project.

One main issue related to the modelling of TGx data is that the studies usually have a smaller number of samples than those needed from the DL methods. Thus, it is more difficult to assess whether the DL model can be well representative of a broad space of samples and if the conclusions that can be drawn from the model are reliable. One solution is to use transfer learning methodology under the assumption that the knowledge learned from a dataset can be used to improve the learning process from a different dataset with limited information [160]. Even if transfer learning has been successfully applied in image or video analysis and speech recognition, few efforts have been made to apply it to TGx studies. For example, Chen et al. [161] developed a multitask multilayer feedforward neural network to inference the gene expression by using LINCS 1000, Genotype-Tissue Expression (GTEx) data and 1000 Genomes expression. Furthermore, DL methods have been successfully applied in the context of de novo drug design. For example, Popova et al. [162] developed a new computational strategy called ReLeaSE (Reinforcement Learning for Structural Evolution) that integrates two deep neural networks, one generative and one predictive, that are used to generate novel target chemical libraries.

### 6.6. Data Integration for Multi-Omics Analyses

The rapid advances of high-throughput “-omics” technologies lead to the production of different kinds of omics data, such as gene expression, microRNA expression (miRNA), copy number variation (CNV), single nucleotide polymorphism (SNP) and protein-protein interactions (PPI). Each of these experimental data potentially provides complementary information about the whole studied organism [163,164].

Depending on the nature of the data and on the statistical problem to address, the integration of heterogeneous data can be performed at different levels: early, intermediate and late [25]. Early integration consists in concatenating data from different views in a single one, without changing the nature of the data. This first type of integrative strategy applied in a TGx study allows us to increase the number of samples related to a particular experimental condition or to compare different experimental results [165,166]. The transcriptomics datasets coming from different studies are first independently preprocessed and then concatenated to form a single dataset [167,168,169]. Intermediate integration consists of transforming all the data sources into a common feature space before combining them. In the late integration methodologies, each view is analyzed separately and the results are then combined. Late integration methods are mainly used to combine statistics p-values across different studies. Different methodologies exist such as the combination of effect size and the Fisher sum of logs method. These methodologies can be strongly influenced by outliers, thus rank-based methodologies have been proposed to obtain more stable results [170,171].

Moreover, different TGx studies integrated gene expression or RNA-Seq data with biological assays, clinical chemistry, therapeutic categories or molecular pathways to get increasingly exhaustive reasoning of biological mechanisms and cellular functions associated with adverse outcomes from environmental exposures and toxicants [172]. For example, Zhang et al. [173] assessed the toxic effect of doses of Zearalenone on cultured donkey granulosa cells (dGCs) by integrating gene expression data from RNA sequence analysis and RT-qPCR and immunofluorescence staining of dGCs, showing the dysregulation of apoptosis-related genes and induction of ovarian cancer-related genes via the PTEN/PI3K/AKT signaling pathway. Scala et al. performed an integrative analysis in which they combined the alterations of DNA methylation, mRNA and microRNA expression of ten carbon nanomaterials in order to better characterize their regulatory and functional map in three human cell lines [3,174].

Furthermore, a different set of ML methods, both supervised and unsupervised have been proposed for multi-omic data analysis. For example, Pavlidis et al. [175] proposed an intermediate integration method based on SVM, to integrate microarray expression and phylogenetic profiles in order to infer gene function. Similarly, Napolitano et al. [9] proposed a methodology for drug repositioning by integrating genome-wide gene expression measures, chemical structure and drug targets. Moreover, Kim et al. developed the Analysis Tool for Heritable and Environmental Network Associations (ATHENA), a grammatical evolution neural networks (GENN) algorithm to integrate different omics data for identifying features associated with cancer clinical outcomes [176]. Different unsupervised multi-view clustering methodologies have been proposed, such as MVDA [25] and SNF [177], for patient stratification in cancer studies or iNMF that is a multi-view biclustering algorithm for module detection genomic datasets [178].

#### Integrate Transcriptomic Datasets with Molecular Descriptors for Hybrid Qsar Models

Following the assumption that the relationship between structural properties and phenotypic effects of exposure is indirectly mediated by its MOA, an alternative approach to identify markers for toxicity could focus on defining hybrid predictive models that combine both structural properties and TGx features [9]. For example, Perualila-Tan et al. combined gene expression and chemical information to infer if the gene expression response is caused by the presence or absence of a particular chemical sub-structure [179]. In addition, Serra et al. proposed a methodology that integrates molecular descriptors and gene modifications to create a hybrid QSAR model that predicts human serum albumin binding of small molecules [180].

## 7. Conclusions

In this third part of the three-article series review on transcriptomics data in TGx, we provided an overview of the state-of-the-art methodologies to analyze, interpret and model TGx data that are used to better explain the compounds’ MOAs and to perform toxicity predictions.

The availability of open source transcriptomics datasets led to the development of different downstream analysis and modelling methodologies to answer specific research questions. For example, the BMD analysis allows identifying the minimal doses that affect the gene expression. The gene co-expression network analysis can elucidate the similarity/dissimilarity in treatment response. Read-across methods can be used to fill data-gaps and translate knowledge from existing compounds to the most similar ones. AOPs can be used to explain the links between a molecular initiating event after exposure and the final adverse outcome by creating a chain of relevant key events. Furthermore, ML methods can be used to create accurate and reliable models for toxicity prediction. Key aspects are robust and accurate predictions, rigorous model validation, well defined AD, and when possible an easy interpretation of model results. Predictive models that satisfy these requirements might assist the risk assessment and decision-making procedure [81].

One major issue of concern is the reproducibility of data and the quality assurance that are of utmost importance for all data used for modelling. Lack of high-quality data will result in unreliable in silico models that will not be exploitable for regulatory purposes. A more thoughtful discussion about data generation can be found in the first part of this review series. Moreover, open data access, open protocols and publicly available meta-data annotations, although not the focus of this review, are integral for reproducible analyses as part of the FAIR Data Principles in order to make data findable, accessible, interoperable and reusable [181].

In conclusion, TGx methodologies have a good potential to become part of the regulatory hazard assessment when all aspects from data generation to data preprocessing and modelling will be harmonized, and openly available for the scientific and regulatory communities. Furthermore, we believe that some methodologies and techniques implemented in other fields (e.g., QSAR) could be translated in the contest of TGx. Eventually, future methods could combine ML algorithms and dose-dependencies methods in order to identify biomarkers of toxicity.

This review can be considered the starting point to identify the best downstream analysis methodology to apply to TGx data depending on the problem in hand. It is important to highlight that each one of the described methods can be used individually, but they can also be concatenated in a pipeline to perform a more comprehensive TGx analysis. Moreover, it is important to note that all the modelling methodologies strongly rely on careful planning of the exposure conditions and robust data preprocessing, discussed in detail in the first and second parts of this review series.

## Figures and Tables

**Table 1:** Tools available for benchmark dose analysis.

	BMDS	PROAST	BMDExpress 2	ISOgene	BMDx
EPA Models *	X				X
Probe id	-	-	X	
Gene id	-	-		X
BMD/BMDL	X	X	X		X
BMDU	X	X			X
IC50					X
EC50					X
Enrichment Analysis	-	-	X		X
Interactive enriched maps	-	-			X
Comparisons at different time points	-	-			X
GUI	X	X	X	X	X

* Models approved by the US Environmental Protection Agency.

## Supplementary Materials

The following are available online at https://www.mdpi.com/2079-4991/10/4/708/s1. Table S1: Some well-known clustering algorithms, including their type of classification, similarity measures that can be used, how the choice of the number of clusters is handled, and algorithm input.

## Abbreviations

The following abbreviations are used in this manuscript:

AD	applicability domain
AI	artificial intelligence
AIC	Akaike criterion
ANNs	artificial neural networks
AOP	adverse outcome pathways
ATHENA	Analysis Tool for Heritable and Environmental Network Associations
BMD	benchmark dose
BMDL	benchmark dose lower bound
BMDU	benchmark dose upper bound
BMR	benchmark regulation
CART	classification and regression trees
CFS	correlation feature selection
CNN	convolutional neural network
CNV	copy number variation
CMAP	Connectivity Map
DAGs	directed acyclic graphs
dGCs	donkey granulosa cells
DL	deep learning
DT	decision trees
EFSA	European Food Safety Authority
FN	false negative
FNN	feedforward neural network
FP	false positive
GCN	graph convolutional network
GENN	grammatical evolution neural network
GFA	group factor analysis
GO	gene ontology
GTEx	Genotype-Tissue Expression
KE	key events
K-NN	k-nearest neighbors
IC50	half maximal inhibitory concentration
L1000	Library of Integrated Network-Based Cellular Signatures 1000
LDA	linear discriminant analysis
LDrA	Latent Dirichlet Allocation
LR	logistic regression
MDS	multidimensional scaling
MF	matrix factorization
MI	mutual information
ML	machine learning
MOA	mechanism of action
MOE	molecular initiating event
MVDA	multi-view data analysis
miRNA	microRNA
MTF	bayesian multi-tensor factorization
NAM	novell assessment methods
NB	naive bayes
OECD	Organisation for Economic Co-operation and Development
Open TG-GATEs	Open Toxicogenomics Project-Genomics Assisted Toxicity Evaluation System
PCA	principal component analysis
PLSDA	partial least squares discriminant analysis
POD	point of departure
PPI	protein-protein interactions
PTGS	Predictive Toxicogenomics Space
QSAR	quantitative structure activity relationship
ReLU	Rectified Linear Unit
RF	random forest
RIVM	Rijksinstituut voor Volksgezondheid en Milieu institute
RNA-Seq	RNA sequencing
SNF	similarity network fusion
SNP	single nucleotide polymorphism
SVM	support vector machines
tSNE	t-distributed stochastic neighbour embedding
TGx	Toxicogenomics
TN	true negative
TP	true positive
UMAP	Uniform Manifold Approximation and Projection
