# Supplementary material for: Transcriptomics in Toxicogenomics, Part III: Data Modelling for Risk Assessment
Source: Nanomaterials (Basel). 2020 Apr 8;10(4):708. doi: 10.3390/nano10040708 (PMC7221955; doi:10.3390/nano10040708)
Supplement: Supplementary file 1 [file nanomaterials-10-00708-s001.pdf]

Supplementary Table S1. We list some well-known clustering algorithms, including their type of classification, similarity measures that can be used, how the choice of the number of clusters is handled, and algorithm input.

**Name**	**Type of classification**	**Similarity concept**	**k**	**Input**
K-means [A comparative study of efficient initialization methods for the k-means clustering algorithm. 2013]	Hard classification	Euclidean distance	k as input	Object coordinates, k
K-medians	Hard classification	Manhattan distance	k as input	Object coordinates, k
Partitioning around medoids (PAM)	Hard classification	User provided similarity	k as input	Similarities, k
Affinity propagation [Clustering by Passing Messages Between Data Points. 2007]	Hard classification	User provided similarity	Chooses k	Similarities, damping factor
Density based [Density-based clustering. 2011]	Hard classification	User provided distance	Chooses k	Neighbours relation, min neighbours for dense region
Spectral clustering [A tutorial on spectral clustering. 2007]	Hard classification	User provided similarity	k as input	Similarities, k
Hierarchical clustering [Assessment of Drugs Toxicity and Associated Biomarker Genes Using Hierarchical Clustering. 2019]	Hard classification	User provided distance	Offers options for each k	Distances
Mixture models [Finite mixture models and model-based clustering. 2010; Model-based clustering of microarray	Probabilistic type (soft classification)	Uses coordinates	Depends on specific algorithm	Object coordinates, perhaps k

expression data via latent Gaussian mixture models. 2010; Model-based clustering of high-dimensional data: A review. 2014]				
Fuzzy clustering [Review on Fuzzy Clustering Algorithms. 2008]	Soft classification	Depends on specific algorithm	Depends on specific algorithm	Depends on specific algorithm
Biclustering (a.k.a. co-clustering) [A systematic comparative evaluation of biclustering techniques. 2017; Biclustering as Strategy for Improving Feature Selection in Consensus QSAR Modeling. 2018; Robust Co-clustering to Discover Toxicogenomic Biomarkers and Their Regulatory Doses of Chemical Compounds Using Logistic Probabilistic Hidden Variable Model. 2018]	Depends on specific algorithm	Depends on specific algorithm	Depends on specific algorithm	Depends on specific algorithm
Consensus clustering [Analyzing High Dimensional Toxicogenomic Data Using Consensus Clustering. 2012]	Hard classification	Uses results from other clusterings	Chooses k	Previous clusterings
